# Arterial anastomosis in LDLT: techniques and risks

**DOI:** 10.1007/s13304-024-01983-4

**Published:** 2024-09-26

**Authors:** Stefano Di Sandro, Barbara Catellani, Deniz Balci, Fabrizio Di Benedetto, The Micro-Surgical Study Group

**Affiliations:** 1 Hepato-Pancreato-Biliary Surgery and Liver Transplantation Unit, University of Modena and Reggio Emilia, Via del Pozzo 71, 41124 Modena, Italy; 2 Department of Surgical, Medical, Dental and Morphological Sciences with Interest Transplant, Oncological and Regenerative Medicine, University of Modena and Reggio Emilia, Modena, Italy; 3 Department of General Surgery and Organ Transplantation, Bahcesehir University School of Medicine, Istanbul, Turkey; 4 Clinical and Experimental Medicine PhD program, University of Modena and Reggio Emilia, Modena, Italy

**Keywords:** Living donor liver transplantation, arterial anastomosis, running suture, interrupted stiches, microscope, loupes

## Abstract

Reconstructing the hepatic artery in adult living donor liver transplantation is technically challenging, with complications leading to significant morbidity and mortality. Early arterial thrombosis can result in a mortality rate up to 50%, often necessitating re-transplantation. The most common techniques for arterial anastomosis include end-to-end reconstruction with interrupted or continuous sutures, either using magnifying loupes or a microscope. Although microscopes provide enhanced precision, they do not significantly reduce early thrombosis rates compared to loupes but increase surgical time. Overall, surgeons can achieve early thrombosis rates below 1% with experience and evolving techniques.

**Supplementary Information:**

The online version contains supplementary material available at 10.1007/s13304-024-01983-4.

## Technical notes

The reconstruction of the hepatic artery in living donor liver transplantation in adult recipients is one of the most technically complex steps [1]. Complications associated with hepatic artery reconstruction are correlated with increased short- and long-term morbidity and mortality in patients who develop them [1, 2]. Generally, patients who develop early unresolved arterial thrombosis have a mortality rate that reaches 50%, and in patients who do not develop severe imminent consequences, the only alternative is re-transplantation. For this reason, over the years, the authors have reported their experiences to share the best reconstruction techniques for the artery, the results associated with them, and the prospect of reducing the incidence of early arterial complications below the threshold of one percent [3].

Based on this evidence, the three most widespread techniques in the world for arterial anastomosis in living donor liver transplantation are: end-to-end reconstruction with interrupted sutures using magnifying loupes, continuous suture reconstruction with magnifying loupes, or the same reconstructions performed with the aid of a magnifying microscope [4–6]. In the video attached to this manuscript, we demonstrate the different approaches most used in centers and reported in the literature through executed cases.

The first case deals with an end-to-end reconstruction with interrupted sutures using a 9/0 prolene thread. Two micro bulldogs are placed on the proximal stump (recipient side) and distal stump (graft side) of the artery. The suture begins with the placement of a first stitch at 3 o’clock and a second stitch at 9 o’clock. Subsequently, interrupted stitches are placed starting from the 3 o’clock corner until reaching the 9 o’clock corner. Each stitch is placed by passing the needle from the inside to the outside of the arterial stump walls. Once the anterior wall suture is completed, the stitches are tied without knotting the corners. After this phase, the artery is rotated to similarly suture the posterior wall. Usually, the posterior wall is knotted after removing the bulldog from the distal stump. The corners are knotted at the end after also removing the bulldog from the proximal stump and reperfusing the artery.

The second technique consists of a continuous suture anastomosis. Usually, an 8/0 or 9/0 prolene thread is used. As demonstrated in the attached video, the most frequently adopted strategy is called the “parachute” approach, which allows for a better view of the lumen and thus the vessel wall to be sutured. In addition, it is easier and more precise to define the distance for passing the continuous suture stitches. At the end of the posterior wall suture, the right and left ends of the suture are gently pulled to approximate the stumps. At this point, once the stumps are approximated, the anterior wall suture is performed starting from the 3 o’clock corner to the 9 o’clock corner, completing the entire anastomosis. The bulldogs are removed, first the proximal and then the distal, and the artery is knotted with flow.

The last approach described is based on a continuous suture anastomosis with the aid of a surgical microscope. The arterial stumps are clamped with two micro bulldogs installed on a spacer. This anastomosis is performed by placing and knotting a prolene 8/0 stitch at the 3 o’clock corner and suturing the anterior wall continuously until 9 o’clock. At this point, the stumps are rotated with the spacer, and the suture is passed under to invert the corners and expose the posterior wall anteriorly. Starting from the 3 o’clock corner, the posterior wall is also sutured continuously until reconnecting with the starting corner, and the continuous suture is knotted with the thread end used for the first corner stitch at the beginning of the anastomosis.

The use of the microscope is highly promoted by some surgical schools, especially Oriental ones [5]. Comparing the results of reconstructions performed with loupes or with a microscope, there does not appear to be a change in the incidence of early arterial thrombosis [4]. However, the surgical time is significantly longer in anastomoses performed with a microscope compared to using loupes. There are various types of loupes, the most commonly used being 3.5 × or 5.0 × magnification. In 2017, Li and collaborators [4] reported their experience of over 700 arterial reconstructions in living donor liver transplants, demonstrating an overall early arterial thrombosis incidence of 1.8%, which dropped to 1.2% in the second era using only surgical loupes instead of the microscope. In 2023, Rela and coworkers [6] even reported an incidence of early arterial thrombosis below 1% thanks to a technique with interrupted stitches described in their manuscript.

In conclusion, the various known techniques demonstrate safety and efficacy without a clear superiority of one technique over another. The possibility of using the microscope should be considered especially for very small caliber arteries, although better incidence results are not known. Surgeons with extensive experience in transplant surgery and microsurgery can achieve excellent results with early arterial thrombosis incidences even below one percent. The technical and technological evolution in this field ensures excellent results for adult recipients of living donor liver transplantation.

## Supplementary Information

Below is the link to the electronic supplementary material.

Supplementary file1 (MP4 954088 KB)

## Data Availability

Statement is not applicable because there are no data reported.
